# Certolizumab-Induced Liver Injury in Ankylosing Spondylitis: A Case Report and Causality Assessment

**DOI:** 10.7759/cureus.66569

**Published:** 2024-08-10

**Authors:** Imane Bensaghir, Latifa Tahiri, Sara Farih, Hanan Rkain, Fadoua Allali

**Affiliations:** 1 Department of Rheumatology B, Ayachi Hospital, Ibn Sina Hospital Center, Faculty of Medicine and Pharmacy, Mohammed V University, Rabat, MAR; 2 Department of Exercise Physiology and Autonomous Nervous System, Faculty of Medicine and Pharmacy, Mohammed V University, Rabat, MAR

**Keywords:** drug-induced liver injury (dili), "ankylosing spondylitis", clinical case report, tumor necrosis factor-alpha (tnf-α) inhibitors, s: hepatotoxicity, certolizumab

## Abstract

Certolizumab-induced liver injury is exceptionally rare, with only a few cases reported in the literature. We present the case of a 34-year-old man with axial ankylosing spondylitis (AS) who developed a drug-induced liver injury following treatment with certolizumab. Despite the initial ineffectiveness of non-steroidal anti-inflammatory drugs and an inadequate response to infliximab, the patient achieved remission of AS symptoms with certolizumab. However, he subsequently developed elevated liver enzymes indicative of hepatocellular injury. Investigations excluded viral hepatitis and autoimmune liver diseases, pointing to certolizumab as the likely cause. The updated Roussel Uclaf Causality Assessment Method confirmed a probable causal relationship between certolizumab and hepatotoxicity. Discontinuation of certolizumab led to normalization of liver enzymes without recurrence of liver injury. This case highlights the need for vigilant monitoring for hepatotoxicity in patients receiving tumor necrosis factor inhibitors.

## Introduction

Ankylosing spondylitis (AS) is a chronic inflammatory rheumatic disease that primarily affects the spine and sacroiliac joints. It is characterized by chronic back pain and morning stiffness, which can progress to spinal fusion. Diagnosis is based on clinical features such as early onset (before age 45), supported by laboratory findings like the HLA-B27 gene and elevated inflammatory markers. Imaging studies, including X-rays and MRIs, can confirm sacroiliitis [1]. Treatment typically involves non-steroidal anti-inflammatory drugs (NSAIDs), with tumor necrosis factor inhibitors (TNFis) used for cases that are refractory to NSAIDs [2]. We present a case of certolizumab-induced liver injury in a patient with AS, highlighting the diagnostic challenges and management considerations associated with this rare complication.

## Case presentation

A 34-year-old man with no significant medical history was diagnosed with axial AS based on the Assessment of SpondyloArthritis International Society classification criteria. He presented with chronic inflammatory pain in the cervical, thoracic, and lumbar spine, lasting for 10 years, and radiographic evidence of sacroiliitis. A physical examination revealed limitations in cervical and lumbar spine mobility, hip pain, and restricted movement.

The patient was refractory to optimal doses of NSAIDs, including diclofenac 150 mg/day, meloxicam 15 mg/day, and indometacin 150 mg/day. Following negative screening for tuberculosis, including negative interferon gamma release assays, tuberculin skin tests, and normal liver and kidney tests, treatment with intravenous infliximab was initiated at a dosage of 5 mg/kg at weeks 0, 2, 6, and then every eight weeks.

Despite five months of infliximab therapy, the patient’s symptoms persisted, with a Bath Ankylosing Spondylitis Disease Activity Index of 4 and an Ankylosing Spondylitis Disease Activity Score with CRP of 3.8, along with elevated CRP levels (37.4 mg/L). Consequently, the decision was made to switch the patient to certolizumab, administered subcutaneously at a dose of 400 mg at weeks 0, 2, 4, and then every four weeks.

After six months of certolizumab therapy, the patient achieved remission of AS symptoms but developed elevated liver enzymes: aspartate aminotransferase (AST) and alanine aminotransferase (ALT), with AST two times and ALT three times the normal range, without associated symptoms (Table 1).

**Table 1:** Disease activity assessment during infliximab and certolizumab therapy ASDAS-CRP: Ankylosing Spondylitis Disease Activity Score with CRP; BASDAI: Bath Ankylosing Spondylitis Disease Activity Index

Parameter	At five months of infliximab	At six months of certolizumab	Reference range
CRP	37.4	2.1	<5 (mg/L)
BASDAI	4	0.2	-
ASDAS-CRP	3.8	0.5	-

Investigations

Liver tests revealed elevated AST and ALT levels, indicating a hepatocellular pattern of injury. Serological tests for viral hepatitis were negative, and autoimmune liver antibodies were also negative, including antinuclear antibodies, smooth muscle antibodies, antimitochondrial antibodies, antibodies to liver-kidney microsome type-1, antibodies to soluble liver antigen, and antibodies to asialoglycoprotein receptor. A liver ultrasound showed no evidence of structural abnormalities. The updated Roussel Uclaf Causality Assessment Method (RUCAM) was employed to evaluate the likelihood of certolizumab-induced hepatotoxicity, resulting in a score of six, consistent with probable causality.

Treatment and outcome

Certolizumab therapy was discontinued, and liver enzymes normalized within one month. The patient remained asymptomatic throughout the episode of hepatotoxicity. Reintroduction of NSAIDs did not lead to a recurrence of elevated liver enzymes (Table 2).

**Table 2:** Liver enzyme levels before and after certolizumab discontinuation ALT: alanine aminotransferase; AST: aspartate aminotransferase

Liver enzymes	At 24 weeks of certolizumab therapy	Four weeks after certolizumab discontinuation	Reference range (IU/L)
AST	89	22	5-34
ALT	161	27	0-55

## Discussion

In this case report, the observed hepatocellular liver injury pattern, along with negative results on autoimmune serological tests and improvement upon discontinuation of certolizumab, supports a diagnosis of drug-induced liver injury (DILI). Using the updated RUCAM [3], we evaluated the likelihood of DILI, resulting in a score of six, indicative of a probable causal relationship. Despite the patient’s history of NSAID usage, the elevation of liver enzymes coincided with the initiation of certolizumab, suggesting it was the precipitating factor for hepatocellular injury. Additionally, liver enzyme levels remained within the normal range upon reinitiating NSAIDs during follow-up.

The updated RUCAM score is a well-established and quantitative method for assessing the likelihood that a specific medication caused liver injury. Developed through international consensus meetings, it assigns points based on factors such as the time course of injury after starting the medication, improvement upon discontinuation, and the presence of alternative explanations for liver damage. The updated version of RUCAM offers improved clarity and reduced scoring variability compared to the original method. It is widely used worldwide, having been applied in over 81,856 DILI cases as reported until mid-2020 [3].

While rare, TNFi-induced hepatotoxicity has been documented. A study conducted by Ghabril et al. [4] identified 34 cases of DILI linked to infliximab (n = 26), etanercept (n = 4), and adalimumab (n = 6). This study suggests that DILI may be a class-wide phenomenon among anti-TNF agents. Additionally, recent research on the leading causes of DILI revealed that anti-TNF agents ranked as the third most prevalent cause, occurring in approximately one in 148 patients treated with these therapies [5].

In Iceland, 11 cases of liver injury associated with anti-TNF-α agents were documented. Among these cases, the causality between liver damage and anti-TNF-α treatment was deemed highly probable in eight out of 11 instances [6]. A prospective study in the same country identified 96 patients with DILI between 2010 and 2011. Although the proportion of cases attributed to azathioprine and infliximab was relatively small (4%), these drugs posed the highest risk of liver injury among patients with DILI [5,7].

Currently, DILI has been linked to all anti-TNF-α medications used in clinical settings. Mancini and colleagues conducted an analysis focusing on liver injury associated with infliximab [8]. Infliximab has the potential to induce liver damage through both immune-mediated and direct mechanisms, typically occurring within one to 12 infusions [7]. While liver injury patterns can vary, reports commonly highlight a predominantly hepatocellular pattern, although both hepatocellular and autoimmune patterns can manifest [8-12].

There are only a few cases in the literature reporting certolizumab-induced liver injury. A recent case described a woman in her 30s who showed elevated liver enzymes following the initiation of certolizumab, with an updated RUCAM score of seven. Liver enzymes normalized upon discontinuation of certolizumab [13]. This case, along with our own, highlights the importance of considering certolizumab-induced liver injury as a potential adverse effect, despite its relative rarity.

## Conclusions

Certolizumab, a TNFi, is an effective therapeutic option for patients with AS who are refractory to conventional treatments. However, clinicians should be vigilant about the potential for hepatotoxicity associated with this medication. In our case, the discontinuation of certolizumab led to the resolution of liver injury without recurrence of symptoms, thereby supporting the diagnosis of DILI caused by certolizumab. This case underscores the importance of meticulous monitoring for adverse effects and prompt intervention in patients receiving TNFi therapy for AS. Further research is needed to elucidate the underlying mechanisms and risk factors associated with TNFi-induced hepatotoxicity.
